# Toll-Like Receptor 4 Knockout Mice Are Protected against Endoplasmic Reticulum Stress Induced by a High-Fat Diet

**DOI:** 10.1371/journal.pone.0065061

**Published:** 2013-05-31

**Authors:** Nicolas Pierre, Louise Deldicque, Caroline Barbé, Damien Naslain, Patrice D. Cani, Marc Francaux

**Affiliations:** 1 Institute of Neuroscience, Université catholique de Louvain, Louvain-la-Neuve, Belgium; 2 Department of Kinesiology, Exercise Physiology Research Group, KU Leuven, Belgium; 3 Louvain Drug Research Institute, Université catholique de Louvain, Brussels, Belgium; University of Bari, Italy

## Abstract

The purpose of this study was to investigate whether toll-like receptor 4 (TLR4) is implicated in the development of endoplasmic reticulum stress (ER stress) observed after a high-fat diet (HFD) in liver, skeletal muscle and adipose tissue. TLR4^−/−^ and C57BL/6J wild-type mice (WT) were fed with chow or HFD (45% calories from fat) during 18 weeks. An oral glucose tolerance-test was performed. The animals were sacrificed in a fasted state and the tissues were removed. TLR4 deletion protected from body weight gain and glucose intolerance induced by HFD whereas energy intake was higher in transgenic mice suggesting larger energy expenditure. HFD induced an ER stress in skeletal muscle, liver and adipose tissue of WT mice as assessed by BiP, CHOP, spliced and unspliced XBP1 and phospho-eIF2α. TLR4^−/−^ mice were protected against HFD-induced ER stress. Then, we investigated the main signaling downstream of TLR4 namely the NF-κB pathway, expecting to identify the mechanism by which TLR4 is able to activate ER stress. The mRNA levels of cytokines regulated by NF-κB namely TNFα, IL-1β and IL-6, were not changed after HFD and phospho-IκB-α (ser 32) was not changed. Our results indicate that TLR4 is essential for the development of ER stress related to HFD. Nevertheless, the NFκ-B pathway does not seem to be directly implicated. The reduced fat storage in TLR4^−/−^ mice could explain the absence of an ER stress after HFD.

## Introduction

Obesity is often associated with a chronic low grade inflammatory state [1], [2], which in turn contributes to the development of various metabolic diseases such as insulin resistance [3], [4], [5], [6]. During the last decade, major advances were made for identifying the molecular mechanisms leading to lipotoxicity in various cell types [7]. A high level of circulating non-esterified fatty acids (NEFA) is able to induce insulin resistance within a few hours in healthy volunteers suggesting that an alteration in intracellular lipid metabolism may impair insulin signaling [8], [9]. Ceramides and diacylglycerol (DAG) are lipid-derived toxic metabolites inhibiting insulin signal via dephosphorylation of protein kinase B (PKB) and phosphorylation of a serine residue on insulin receptor substrate (IRS) [10], [11]. However, one may not exclude that circulating NEFA *per se* are able to act on insulin sensitivity through binding to a membrane receptor and activating a downstream cascade of events that would crosstalk with insulin signaling. Indeed, previous observations suggest that circulating NEFA can bind a plasma membrane receptor, namely the toll-like receptor 4 (TLR4), and its co-receptors CD14 and MD-2 [12], [13].

The TLR are members of the pattern-recognition receptor family that play a key role in the innate immune system [14]. To date, thirteen mammalian TLR have been identified [14]. TLR4, a subclass of TLR, is activated by the lipidic domain of lipopolysaccharide (LPS), a gram-negative bacterial cell wall component [15]. Ablation of TLR4 in transgenic mice confers partial protection against insulin resistance induced by lipid infusion or high-fat diet (HFD) [12], [16], [17], [18], [19], [20]. Therefore, TLR4 could be the missing link between high circulating lipids and insulin resistance.

Lipid excess is known to cause another cell stress known as endoplasmic reticulum (ER) stress [21], [22], [23], [24] which is the consequence of a disequilibrium between the folding capacity of ER and the amount of proteins to be folded. To cope with this stress, the cell triggers a series of signal transduction known as the unfolded protein response (UPR), which is initiated at the level of three ER stress sensors: inositol-requiring 1α (IRE1α), double-stranded RNA-dependent protein kinase (PKR)-like ER kinase (PERK) and activating transcription factor 6 (ATF6). Each of these sensors is associated with the protein chaperone immunoglobulin-heavy-chain-binding protein (BiP). Upon accumulation of unfolded proteins in the ER lumen, BiP is released from PERK, IRE1α and ATF6 leading to their activation and downstream signaling. These pathways initiate a homeostatic response via: 1) protein synthesis inhibition through the phosphorylation of eukaryotic translation-initiation factor 2α (eIF2α); 2) transcriptional up-regulation of genes involved in protein folding, transport and ER-associated protein degradation; 3) apoptosis mediated by C/EBP homologous (CHOP) [23].

ER stress was observed in liver of ob/ob and high-fat fed mice and was proposed to be involved in the pathophysiology of insulin resistance through an interaction between UPR and inflammation signaling [25]. Indeed, a growing body of evidence suggests that UPR and insulin resistance are interconnected through at least 2 mechanisms: 1) IRE1α pathway induces c-Jun N-terminal kinase (JNK) phosphorylation which inhibits IRS; 2) PERK and IRE1α pathway activate transcription factors such as nuclear factor-κB (NF-κB) and activator protein 1 (AP-1) which migrate to the nucleus and induce the transcription of cytokines known to contribute to the development of insulin resistance [23], [25], [26].

ER stress and TLR4 signaling are both major players in the development of insulin resistance induced by lipid excess. Whether those two actors act independently or are causally linked is still unknown. In the present study, we hypothesized that TLR4 signaling would mediate lipid-induced ER stress via activation of the NF-κB pathway. To test this hypothesis, TLR4 knockout (TLR4^−/−^) and wild-type (WT) mice were fed with a HFD and the activation of ER stress in liver, skeletal muscle and adipose tissue was measured. Our results show that ER stress is activated by a HFD in WT whereas it is not in TLR4^−/−^ mice.

## Materials and Methods

### Animal Care and Protocol

All protocols were approved by the ethical committee for animal use of the Université catholique de Louvain (Belgium) and the housing conditions were as specified by the Belgian Law of April 6, 2010 on the protection of laboratory animals (agreement n° LA 1220548). 14–16-week-old male TLR4^−/−^ [27] and C57BL/6J wild-type mice (WT) were purchased from Transgenose Institute (CNRS, Orléans, France) and the Laboratory of Experimental Surgery (UCL, Woluwé, Belgium), respectively. TLR4^−/−^ genotype was confirmed by PCR. Animals were housed in cages *per* 4–5 in a controlled environment (22–23°C, 14/10 h light/dark cycle). Mice were fed *ad libitum* with one of the two experimental diets during 18 weeks: standard chow (chow) or high-fat diet (HFD). They were divided in four groups: WT mice fed with chow (n = 10) or HFD (n = 9), TLR4^−/−^ mice fed with chow (n = 9) or HFD (n = 8). Standard chow (10% calories from fat, 4% calories from sucrose [28]) and HFD (45% calories from fat, 17% calories from sucrose) were purchased from SAFE (A04, Augy, France) and Research Diets (D12451, New Brunswick, USA), respectively. Food intake was recorded weekly during the 18 weeks of the nutritional intervention. At the end of the protocol, 6-h-fasted mice were anesthetized with an intraperitoneal injection of a solution (4 ml.kg^−1^) containing ketamine (40 mg.ml^−1^) and xylazine (4 mg.kg^−1^) to preserve organ perfusion during dissection. The depth of anaesthesia was assessed by the absence of eyelid and pedal withdrawal reflexes. The right and left gastrocnemius muscles, the left lobe of the liver, the subcutaneous and visceral adipose tissues were rapidly removed and immediately frozen in liquid nitrogen. At the end of the procedure, mice were killed by cervical dislocation. Samples were stored at −80°C until processed.

### Non-esterified Fatty Acids Measurement

Blood samples were taken from portal vein and frozen at −20°C. NEFA were measured by colorimetric assay according to the manufacturer’s guidelines (Diagnostic Systems, Holzheim, Germany).

### Protein Extraction and Western Blotting

Liver (∼50 mg), gastrocnemius (∼50 mg) and subcutaneous adipose tissue (∼100 mg) were ground using a mortar and a pestle (Bel-Art Products, Pequannock, NJ, USA). Liver and gastrocnemius were homogenised in ice-cold and pH 7.0 buffer [20 mM Tris, 270 mM sucrose, 5 mM EGTA, 1 mM EDTA, 1% Triton X-100, 1 mM sodium orthovanadate, 50 mM sodium β-glycerophosphate, 5 mM sodium pyrophosphate, 50 mM sodium fluoride, 1 mM 1,4-dithiothreitol (DTT), and 10% protease inhibitor cocktail 10X (Roche Applied Science, Vilvoorde, Belgium)]. Subcutaneous adipose tissue was homogenised in ice-cold pH 7.4 RIPA buffer [50 mM Tris-HCl, 150 mM NaCl, 1 mM EDTA, 1% NP-40, 0.25% deoxycholic acid, 2 mM sodium orthovanadate, 5 mM phenylmethylsulfonyl fluoride and a protease inhibitor cocktail (Roche Applied Science)]. Homogenates were centrifuged at 10,000 *g* for 10 min, at 4°C. Supernatants were immediately stored at −80°C. Protein concentration was determined using the DC protein assay kit (Bio-Rad Laboratories, Nazareth, Belgium) with bovine serum albumin as a standard. Proteins lysates (40–60 µg) were combined with Laemmli buffer and separated by SDS-PAGE (10–12%) for 1 h at a constant intensity of 40 mA. Proteins were then transferred to PVDF membranes for 2–3 h at constant voltage of 80 V. Membranes were blocked for 1 h in Tris-buffered saline with 0.1% Tween 20 (TBST) containing 5% non-fat dried milk. Then, membranes were incubated overnight at 4°C in TBST containing 1% bovine serum albumin and one of the following primary antibodies (1∶500 or 1∶1000 dilution): BiP, phosphorylated-eIF2α (p-eIF2α, Ser 51), phosphorylated-JNK (Thr183/Tyr185), phosphorylated ERK1/2 (Thr202/Tyr204), phosphorylated p-38 (Thr180/Tyr182), IκB-α, phosphorylated IκB-α (Ser 32) and eEF2 (eukaryotic elongation factor 2). All primary antibodies were obtained from Cell Signaling Technology (Leiden, The Netherlands). Membranes were washed three times with TBST then incubated 1 h at room temperature with a secondary antibody (1∶10,000 or 1∶5,000 diluted in 5% non-fat dry milk) conjugated to horseradish peroxidase (Sigma, Bornem, Belgium). After three additional washes, chemiluminescent detection was carried out using an ECL Western blotting kit (Amersham ECL Plus, GE Healthcare, Diegem, Belgium). Pictures were taken with a charge-coupled device (CCD) camera (Gbox, Syngene, The Netherlands). Signal quantification was determined by GeneTool software (Syngene). All results were normalized to eEF2 protein then expressed relative to the control group (WT mice fed with chow).

### RNA Extraction and Quantitative Real-time PCR

Total RNA extraction from frozen gastrocnemius (∼50 mg), liver (∼30 mg) and subcutaneous adipose tissue (∼70 mg) was done with Trizol® (Invitrogen, Vilvoorde, Belgium), according to the manufacturer’s instructions. The RNA quality and quantity were assessed by 1.5% agarose gel electrophoresis and Nanodrop® spectrophotometry. Reverse transcription was performed with iScript cDNA synthesis kit (Bio-Rad) from 1 µg total RNA. Real time PCR experiments were done on a MyIQ2 thermocycler (Bio-Rad) using the following conditions: 3 min at 95°C, followed by 35 cycles of 30 s at 95°C, 30 s at 60°C and 30 s at 72°C. Samples were analyzed in duplicate in 10 µl reaction volume containing 4.8 µl IQSybrGreen SuperMix (Bio-Rad), 0.1 µl of each primer (100 nM final) and 5 µl cDNA. Primers sequences are reported in Table 1. Melting curves were systematically analyzed to ensure the specificity of the amplification process. Target genes were normalized using three reference genes according to geNorm analysis [29] and finally expressed relatively to the control group. The references genes were ribosomal protein L19 (RPL19), cyclophilin (CPHN) and hypoxanthine phosphoribosyltransferase 1 (HPRT1).

**Table 1:** Sequences of primers (5′–3′).

Genes	Forward	Reverse
**XBP1s**	TGAGAACCAGGAGTTAAGAACACGC	CCTGCACCTGCTGCGGAC
**XBP1u**	TGAGAACCAGGAGTTAAGAACACGC	CACATAGTCTGAGTGCTGCGG
**CHOP**	CCTGAGGAGAGAGTGTTCCAG	CTCCTGCAGATCCTCATACCA
**RPL19**	GAAGGTCAAAGGGAATGTGTTCA	CCTTGTCTGCCTTCAGCTTGT
**CPHN**	CGTCTCCTTCGAGCTGTTTG	CCACCCTGGCACATGAATC
**HPRT1**	AGGCCAGACTTTGTTGGATTT	CAGGACTCCTCGTATTTGCAG
**IL-6**	ACTTCCATCCAGTTGCCTTCT	GAATTGCCATTGCACAACTCT
**IL-1β**	GTCTGAAGCAGCTATGGCAAC	TTTGAAGCTGGATGCTCTCAT
**TNFα**	CCAGACCCTCACACTCAGATCA	CACTTGGTGGTTTGCTACGAC

XBP1s: X-box-binding protein 1 spliced, XBP1u: X-box-binding protein 1 unspliced, CHOP: C/EBP homologous, RPL19: Ribosomal protein L19, CPHN: Cyclophilin, HPRT1: Hypoxanthine phosphoribosyltransferase 1, IL-6: interleukin-6, IL-1β: interleukin-1β TNFα: tumor necrosis factor-α.

### Oral Glucose Tolerance Test

Seventeen weeks after the beginning of the protocol, 6-h-fasted mice were force-fed with a glucose solution (1 g.kg^−1^ glucose, 40% glucose solution). Plasma glucose concentration was determined with a glucose meter (Roche Diagnostics) on 3.5 µl of blood collected from the tip of the tail vein, 30 min before and 0, 15, 30, 60, 90 and 120 min following glucose gavage. Areas under the curve (AUC) were calculated according to the trapeze method.

### Statistical Analysis

Results are presented as means ± SEM. Two-way ANOVA was used to assess statistical differences amongst means. When appropriate, the Bonferroni test was used as a post-hoc analysis. The significative threshold was set for a *P*-value <0.05.

## Results

### TLR4 Deficiency Protects from Obesity Due to 18-weeks High-fat Diet without Reducing Energy Intake

**Figure 1:**
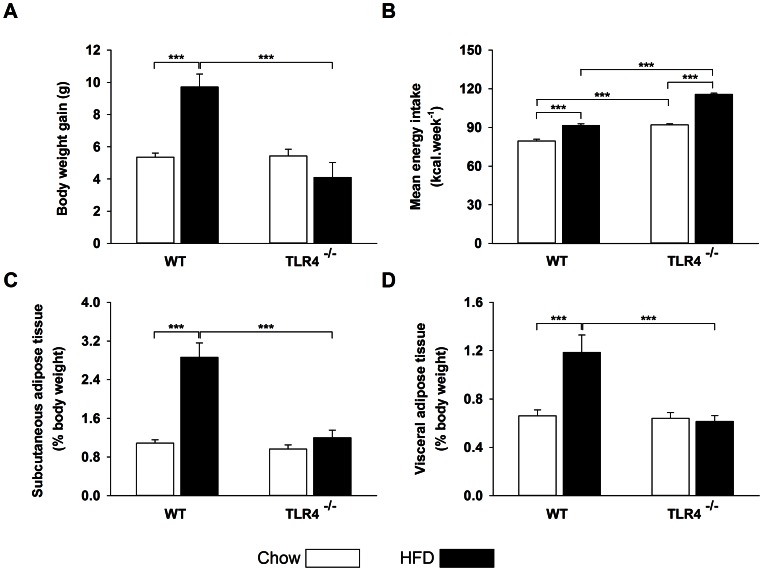
TLR4 deficiency protects from obesity without reducing energy intake. (A) Body weight gain calculated as the body weight difference between the end (18 weeks) and the beginning of the diet. (B) Mean energy intake expressed in kcal.week^−1^ (C) Subcutaneous adipose tissue weight expressed in percentage of body weight. (D) Visceral adipose tissue weight expressed in percentage of body weight. Values are expressed as means ± SEM (n = 8–10), * *P*<0.05, ** *P*<0.01, *** *P*<0.001.

In basal conditions, body weight of TLR4^−/−^ was higher than WT mice (∼2.5 g, *P*<0.001). After 18-weeks chow, body weight was increased by 5.4±0.3 g in WT and 5.4±0.4 g in TLR4^−/−^ mice (Figure 1A). This gain was 81% higher upon HFD in comparison with chow in WT (*P*<0.001) but was not different in TLR4^−/−^ mice (Figure 1A). Compared to chow, visceral and subcutaneous adipose tissues were larger after HFD in WT (79% and 160% respectively, *P*<0.001) but not in TLR4^−/−^ mice (Figure 1C and 1D). When fed *ad libitum* with chow, TLR4^−/−^ mice ingested more energy than WT (12.6 kcal.week^−1^, P<0.001) (Figure 1B). Upon HFD, the increase in weekly energy intake was larger in TLR4^−/−^ mice (26%) than in WT (15%, *P*<0.001) (Figure 1B). All together these results suggest that the energy expenditure of TLR4^−/−^ is higher than WT mice.

### TLR4 Deficiency Protects from ER Stress Induced by a High-fat Diet

**Figure 2:**
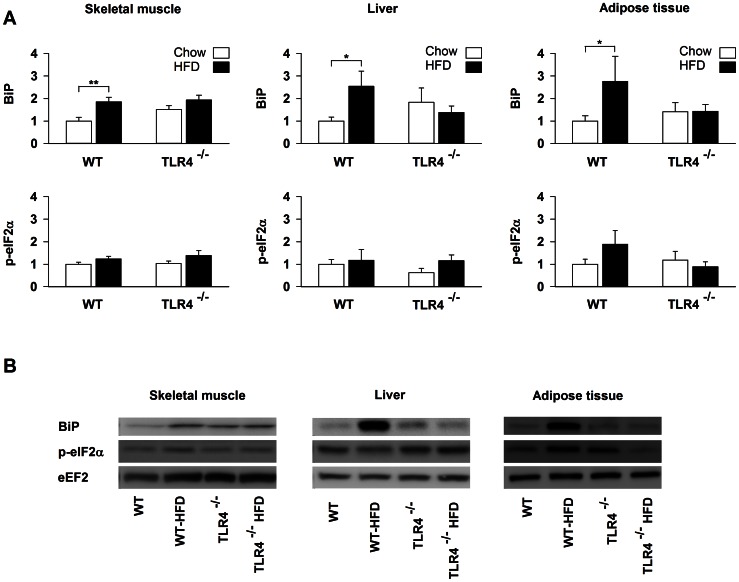
ER stress protein markers are not induced in TLR4^−/−^ mice after a high-fat diet. (A) Protein expression of BiP and phosphorylated eIF2α in skeletal muscle, liver and subcutaneous adipose tissue of wild-type (WT) and TLR4 knockout (TLR4^−/−^) mice fed with a standard (chow) or a high-fat diet (HFD). Results are presented as means ± SEM (n = 8–10), * *P*<0.05, ** *P*<0.01, *** *P*<0.001. (B) Illustration of the data presented in panel A.

BiP expression increased in skeletal muscle (85%, *P* = 0.002), liver (150%, *P* = 0.031) and adipose tissue (188%, *P* = 0.039) of WT mice fed with a HFD, whereas TLR4^−/−^ mice were completely resistant to HFD-induced BiP expression (Figure 2).

**Figure 3:**
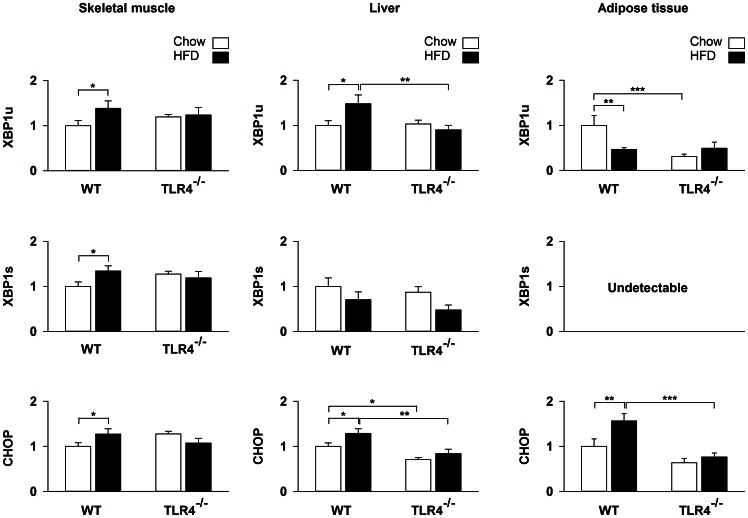
ER stress mRNA markers are not up-regulated in TLR4^−/−^ mice after a high-fat diet. mRNA level of XBP1u (X-box-binding protein 1 unspliced), XBP1s (X-box-binding protein 1 spliced) and CHOP (C/EBP homologous) in skeletal muscle, liver and subcutaneous adipose tissue of wild-type (WT) and TLR4 knockout (TLR4^−/−^) mice fed with a standard (chow) or a high-fat diet (HFD). Results are expressed as means ± SEM (n = 8–10), * *P*<0.05, ** *P*<0.01, *** *P*<0.001.

The unspliced form of XBP1 mRNA (XBP1u) increased by 38% (*P* = 0.041) in skeletal muscle and by 48% (*P* = 0.01) in liver of WT mice upon HFD (Figure 3). Unexpectedly, XBP1u transcripts decreased (−47%, *P* = 0.008) in adipose tissue of WT mice upon HFD (Figure 3). The spliced form of XBP1 mRNA (XBP1s) increased by 34% (*P* = 0.026) in skeletal muscle of WT mice upon HFD (Figure 3). CHOP transcripts were up-regulated by HFD in skeletal muscle (27%, *P* = 0.041), liver (29%, *P* = 0.016) and adipose tissue (56%, *P* = 0.006) of WT mice (Figure 3). HFD did not induce any change in ER stress markers in TLR4^−/−^ mice (Figure 2 and Figure 3).

### TLR4 Downstream Signaling after a High-fat Diet

**Figure 4:**
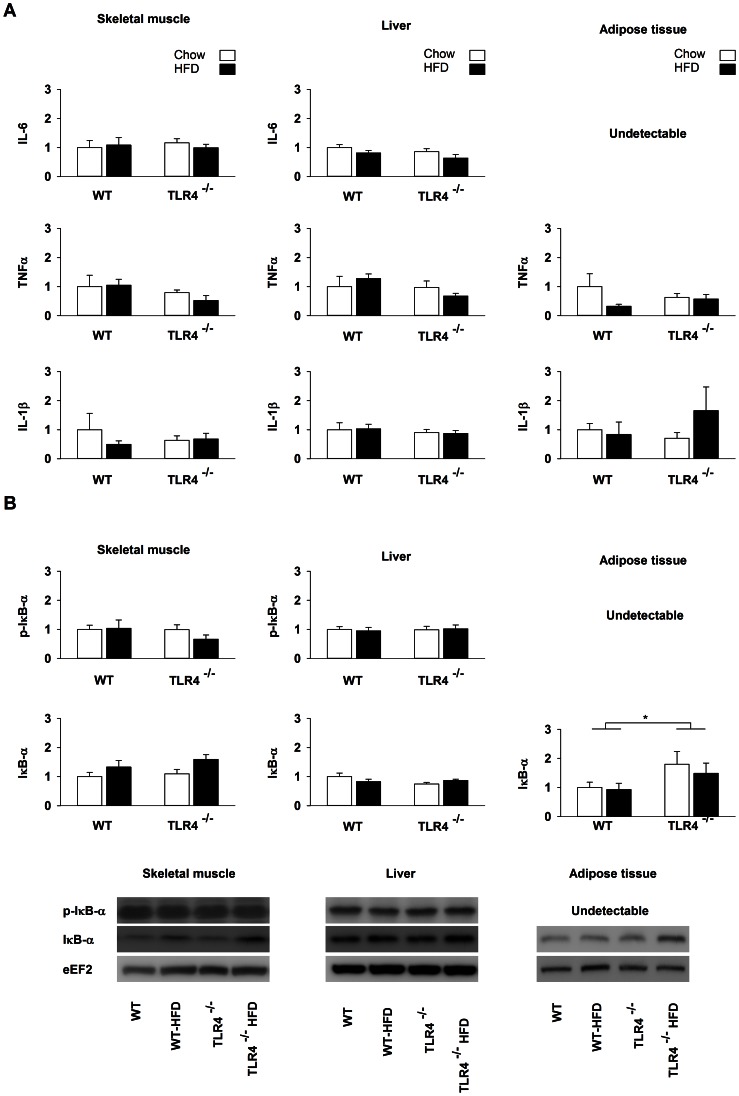
NFκB pathway is not implicated in HFD-induced ER stress. (A) mRNA level of IL-6 (interleukin-6), IL-1β (interleukin-1β), TNFα (tumor necrosis factor-α) are measured in skeletal muscle, liver and subcutaneous adipose tissue of wild-type (WT) and TLR4 knockout (TLR4^−/−^) mice fed with a standard (chow) or a high-fat diet (HFD) (B) Phosphorylation state and expression of IκB-α in skeletal muscle, liver and subcutaneous adipose tissue of wild-type (WT) and TLR4 knockout (TLR4^−/−^) mice fed with a standard (chow) or a high-fat diet (HFD). Results are presented as means ± SEM (n = 8–10), * *P*<0.05, ** *P*<0.01, *** *P*<0.001.

Since our results suggested that ER stress induced by a HFD is dependent upon TLR4, we decided to investigate the signaling downstream of TLR4 expecting to identify the mechanism by which TLR4 is able to activate ER stress. TLR4 leads to the activation of the NF-κB pathway, which regulates the transcription of cytokines such as IL-6, TNFα and IL-1β. Inflammation is known for inducing ER stress [23]. None of the cytokines that we measured was increased after HFD (Figure 4A). Furthermore, the HFD did not increase the phosphorylation state of IκB-α and did not decrease IκB-α protein level (Figure 4B). Nevertheless, in the adipose tissue, IκB-α was 70% higher in TLR4^−/−^ than in WT mice as evidenced by the significant between-strain effect of the ANOVA design (*P* = 0.027). In the same tissue, the level of phospho-IκB-α was undetectable in all conditions.

**Figure 5:**
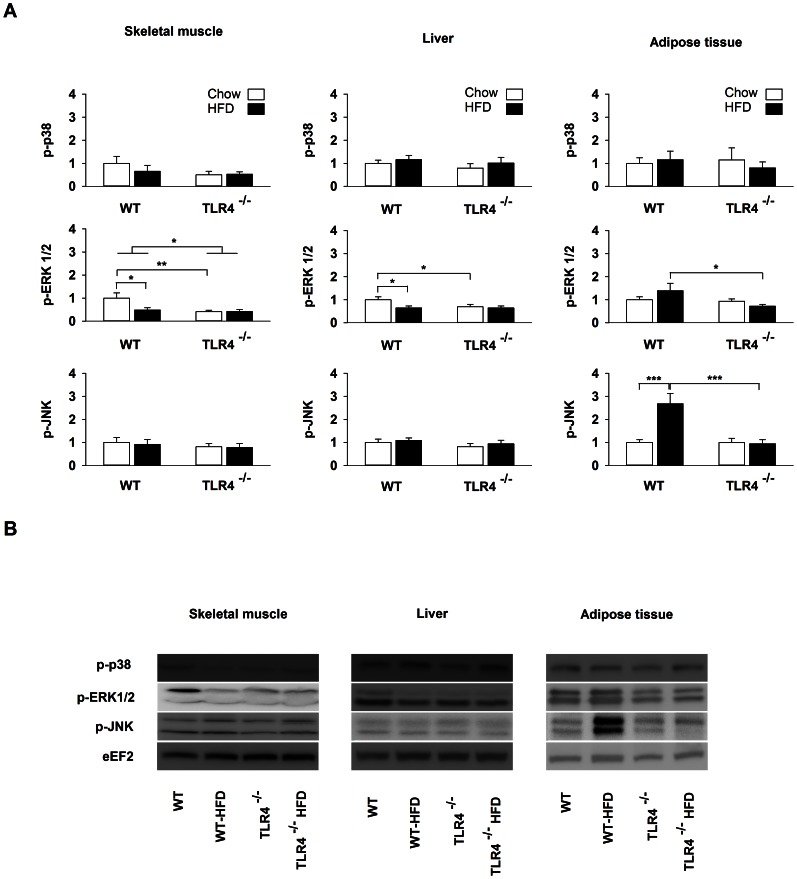
Changes in MAPK phosphorylation after a high-fat diet in wild-type and TLR4^−/−^ mice. (A) Phosphorylation state of p38, JNK and ERK1/2 in skeletal muscle, liver and subcutaneous adipose tissue of wild-type (WT) and TLR4 knockout (TLR4^−/−^) mice fed with a standard (chow) or a high-fat diet (HFD). Results are presented as means ± SEM (n = 8–10), * *P*<0.05, ** *P*<0.01, *** *P*<0.001. (B) Illustration of the data presented in panel A.

The phosphorylation state of the mitogen-activated protein kinases (MAPK) is under the control of the TLR4 pathway [30]. HFD did not change the phosphorylation state of p-38 in all investigated organs (Figure 5). In skeletal muscle, the phosphorylation state of ERK1/2 was 44% lower in TLR4^−/−^ than in WT mice (between-strain effect: *P* = 0.033). The phosphorylation state of ERK1/2 decreased in skeletal muscle (−51%, *P* = 0.016) and liver (−35%, *P* = 0.019) of WT mice upon HFD whereas it was not affected in TLR4^−/−^ mice (Figure 5). After HFD, the phosphorylation state of ERK1/2 was 95% higher in adipose tissue of WT than TLR4^−/−^ mice (*P* = 0.025) (Figure 5). The phosphorylation state of JNK did not change in skeletal muscle and liver, whereas it was dramatically increased by 169% in adipose tissue of WT mice upon HFD (*P*<0.001) (Figure 5). The phosphorylation state of JNK remained unaffected in TLR4^−/−^ mice (Figure 5). These results emphasize the fact that HFD regulates MAPK activity mainly at the level of the adipose tissue.

### Oral Glucose Tolerance Test and Plasma NEFA

**Figure 6:**
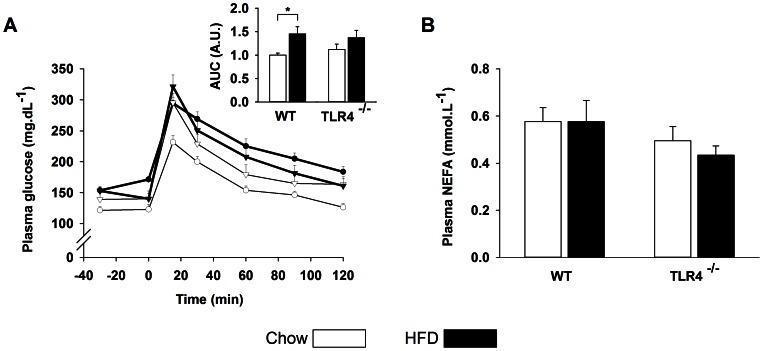
OGTT and circulating NEFA after a high-fat diet in wild-type and TLR4^−/−^ mice. (A) Plasma glucose concentration following an oral glucose load. Wild-type mice upon chow (○), wild-type mice upon a high-fatd diet (•), TLR4 knockout mice upon chow (▿), TLR4 knockout mice upon a high-fat diet (▾). The *inset* represents the area under the curve (AUC) expressed in arbitrary units (A.U.). (B) Fasting NEFA concentrations expressed in mmol.L^−1^.

To assess glucose tolerance, we performed an oral glucose tolerance test (OGTT). After 18-weeks chow, AUC was slightly but not significantly larger in TLR4^−/−^ than in WT mice (Figure 6A). WT mice fed with HFD developed glucose intolerance as demonstrated by a higher AUC (45%, *P* = 0.01), whereas no significant change was observed in TLR4^−/−^ mice (Figure 6A). The circulating NEFA measured in a fasted state were not significantly lower in TLR4^−/−^ mice and were not affected by the HFD (Figure 6B).

## Discussion

### TLR4 Deficiency Protects against ER Stress Induced by a HFD

The main finding of the present study is that TLR4 deficiency protects against ER stress induced by a HFD. BiP is a chaperone that plays a key role in activating cell response to ER stress and its expression is highly regulated by the UPR [31]. In WT mice, BiP was increased during HFD in skeletal muscle, liver and adipose tissues whereas TLR4^−/−^ mice did not show any change. The three branches of the UPR are able to regulate BiP, which is therefore considered as the endpoint marker of the UPR whatever the activated signaling cascade. Consequently, our results confirm the presence of an ER stress in skeletal muscle, liver and adipose tissues of WT mice after a HFD [24], [25]. BiP was unaffected in TLR4^−/−^ mice fed with a HFD suggesting that this receptor contributes to the development of ER stress induced by lipid excess.

CHOP is another well-recognized ER stress marker, which is coordinately regulated with BiP [32]. CHOP is a family member of the ER stress-inducible genes, the transcription of which is regulated both by ATF6 and ATF4, the latter being a downstream effector of the PERK/eIF2α pathway [23]. In WT, CHOP mRNA was increased after a HFD in the three organs analysed in the present study. No changes were observed in TLR4^−/−^ mice supporting the results obtained on BiP.

XBP1u is also a member of the ER stress-inducible gene family, which is regulated by ATF6 [33], [34]. In skeletal muscle and liver, XBP1u mRNA was increased in WT after HFD whereas this change was completely abolished in TLR4^−/−^ mice. These results suggest that the ATF6 pathway was not activated in skeletal muscle and liver of TLR4^−/−^ mice while it was in WT mice. XBP1u seems regulated differently in adipose tissue. Upon chow, XPB1u mRNA was lower in TLR4^−/−^ than in WT mice. It decreased in WT after HFD and remained unchanged in TLR4^−/−^ mice. This clearly indicates that the regulation of the UPR by a HFD is tissue-specific.

XBP1s was undetectable in adipose tissue. In all conditions, the values of cycle threshold (Ct) were higher than 35 and therefore, were not accurate enough for interpretation [35]. This was not the case in skeletal muscle wherein XBP1s was increased in WT upon HFD, but not in TLR4^−/−^ mice. This indicates that the IRE1α pathway was likely activated in WT and that this activation was lacking in TLR4^−/−^ mice. No changes were observed in XPBls mRNA in liver of WT and TLR4^−/−^ mice. This provides an additional argument for a tissue-specific regulation of the UPR by a HFD [36].

We measured the phosphorylation state of eIF2α as a marker of the PERK pathway activation. In our experimental conditions, phospho-eIF2α was affected neither by the mice strain, nor by the diet.

In WT mice fed with HFD, several ER stress markers were increased while others did not change. A distinction must be made in the interpretation of ER stress marker changes. On one hand, the phosphorylation of eIF2α and the splicing of XBP1 are direct markers of UPR activation [37]. On the other hand, BiP, CHOP and unspliced XBP1 are called ER stress-inducible genes, meaning that they are the consequence of UPR. They represent a long term adaptation to a stress condition and may be continuously overexpressed to cope with ER stress. On the contrary, XBP1 splicing and eIF2α phosphorylation seem to be transiently activated. For example, the activation of the PERK pathway is dependent on the acute nutritional status [38]. Oyadomari *et al.* (2008) shown that the phosphorylation of eIF2α is barely detectable in the liver of fasted animals and increased 4 hours after feeding standard diet; this effect was higher in animals fed with HFD. Since the animals used in the present experiment were sacrificed after a fasted period of 6 hours, it is possible that we missed transient changes in the phosphorylation state of eIF2α. Furthermore, caution must be taken when interpreting result from eIF2α phosphorylation as a consequence of ER stress. Beside the control of PERK, eIF2α can also be phosphorylated by three other kinases, namely PKR (protein kinase R), GCN2 (general control nonrepressed 2), HRI (Heme-regulated inhibitor kinase) and dephosphorylated by GADD34 (Protein phosphatase 1 regulatory subunit 15A) [39], [40], [41].

### NFκB Pathway is not Implicated in HFD-induced ER Stress

The results of the present study support the presence of interdependency between TLR4 and ER stress. We tried to further understand how the absence of TLR4 could down-regulate ER stress. Our first hypothesis was based on a reduced activation of the NF-κB pathway in TLR4^−/−^ mice after a HFD, which would reduce the inflammatory state and consequently ER stress. This hypothesis was not corroborated as the NF-κB pathway was not activated after a HFD in WT and TLR4^−/−^ mice in our conditions. The mRNA level of cytokines regulated by NF-κB, namely TNFα, IL-1β and IL-6 [23], was not changed and IκB-α, an inhibitory protein of NF-κB, was not decreased after HFD and it phosphorylation state was not affected.

It has been reported that circulating LPS, the main TLR4 ligand, was increased after a HFD likely because of a more leaky gut [42], [43], [44]. Studies showed that LPS induced ER stress in lung [45], human B cells [46] and liver [47]. Plasma LPS was not measured in the present study. Therefore, we may not rule out that circulating LPS induced ER stress in WT and not in TLR4^−/−^ mice. Nevertheless, this hypothesis seems unlikely because of the lack of NF-κB activation in TLR4^−/−^ and WT mice.

*In vitro* and *in vivo* studies suggest that TLR4 recognizes NEFA in addition to LPS [12], [13]. Like others [48], [49], we did not observe any difference in plasma NEFA concentration after a HFD arguing against a NEFA-dependent activation of TLR4 during a HFD. Nevertheless, the blood samples were taken in a fasted state and therefore, we may not exclude a transitory increase of circulating NEFA during the postprandial phase.

### TLR4 Deficiency Protect against Body Weight Gain and Adipose Tissue Extension Induced by a HFD

In addition to the protection from ER stress, others and we observed that the deletion of the gene coding for TLR4 provides also a protection against obesity [16], [17], [50], probably because of a more favorable energy balance. In parallel to body weight gain, we and others showed that TLR4 deficiency protects from visceral and subcutaneous adipose tissue expansion induced by a HFD [16], [17], [19]. Upon chow, the energy intake was higher in TLR4^−/−^ than in WT mice whereas the body weight gains were similar. Upon HFD, both genotypes increased energy intake, which was the largest in TLR4^−/−^ mice as previously reported [17], [19]. Since the body weight gain was lower in TLR4^−/−^ than in WT mice, it is likely that fat absorption and/or energy expenditure were altered. To the best of our knowledge, no results have been reported regarding an impaired fat absorption in TLR4 deficient mice submitted to a HFD. Indirect calorimetry measurements demonstrated that energy expenditure and fatty acids utilization were increased upon a HFD in mice with a loss of function mutation of the gene coding for TLR4 (C3H/HeJ) [17]. Frisard *et al*. (2010) showed that the skeletal muscle of C3H/HeJ mice exhibits an increased fatty acid oxidation, citrate synthase activity and beta-hydroxyacyl CoA dehydrogenase activity [51]. Therefore, the metabolic consequences related to fat storage could explain the presence of an ER stress in WT after a HFD without a direct implication of the NF-κB pathway. In TLR4^−/−^ mice, the metabolic changes towards greater energy expenditure and reduced body fat content would be protective.

### TLR4 Deficiency Protects from Glucose Intolerance Induced by a HFD

Recent findings showed that high glucose level induces ER stress, probably via the glucosamine pathway [52], [53]. Indeed, an elevated glucose level causes ER stress in primary cardiomyocytes [54], endothelial cells [55], liver [56] and adipocytes [57]. Glucose level was slightly increased in the fasted state after HFD, but the concentrations were not different between WT and TLR4^−/−^ mice. In the present study, glucose tolerance was assessed by an OGTT test. In the chow condition, there was no statistical difference between WT and TLR4^−/−^. After having received a HFD, WT mice became glucose intolerant whereas no difference was observed in TLR4^−/−^ mice. This confirms other studies showing that TLR4 deficient mice are protected from insulin resistance induced by lipid infusion or a HFD [12], [16], [17], [18], [19], [20]. High sucrose diet has also been linked to insulin resistance [58], [59]. In the HFD used in the present experiment, the sucrose content was higher than in chow (17 kcal% *vs* 4 kcal%), but far from the 60–70 kcal% usually used in the high sucrose diets [58], [60]. Therefore, it is likely that ER stress and insulin resistance observed in WT and not in TLR4^−/−^ mice after HFD is mostly due the fat content of the diet even if we may not rule out a partial indirect contribution of sucrose.

In human, TLR4 is more expressed in skeletal muscle of obese type 2 diabetic patients than lean healthy subjects [61]. These results are associated with a positive correlation between TLR4 protein content and insulin resistance, suggesting that TLR4 might be an actor of the process linking inflammation and insulin resistance in mice as well as in human. A broad spectrum of evidence suggests that TLR4 controls its own expression. For example, incubation of human primary myotubes with palmitate or lipid A, the LPS-binding domain of TLR4, increases its expression [61]. On the contrary, TLR4 mRNA is reduced in skeletal muscle of frail obese elderly patients after 12 weeks exercise training [62]. Endoplasmic reticulum dysfunction due to lipid excess is also known to disrupt insulin signal in mice [25]. In human, ER stress was observed in liver and adipose tissue of obese patients [63], [64] and it was reduced after weight loss [65]. All together these results and those reported in the present paper suggest that a down-regulation of TLR4 or a reduced stimulation of this receptor by nutritional, pharmacological and/or exercise interventions could reveal a novel strategy for reducing low grade inflammation and ER stress-induced insulin resistance.

### TLR4 Deficiency Protects from HFD-induced JNK Activation in Adipose Tissue

TLR4 appears to mediate the signal linking obesity to insulin resistance. However, the underlying mechanisms are unclear and seem to be organ-dependent [66]. In the present study, we found that TLR4 was essential for inducing ER stress in the insulin-sensitive tissues during a HFD. ER stress is now recognized as a central mechanism responsible for insulin resistance [23]. Indeed, activated IRE1α recruits TRAF2 (tumour-necrosis factor-α-receptor-associated factor 2) and triggers JNK phosphorylation [26]. The phosphorylation of JNK mediates in turn the phosphorylation of IRS1 (insulin-receptor substrate 1) on serine residues which is known to inhibit phosphorylation of IRS1 on tyrosine residues causing insulin resistance [5]. In our study, the phosphorylation state of JNK was only affected in adipose tissue of WT mice after HFD whereas this effect was completely abolished in TLR4^−/−^. Consequently, it is likely that TLR4 contributes to JNK phosphorylation through ER stress in adipose tissue. This result supports the idea that the mechanism by which TLR4 mediates insulin resistance is organ-dependent. XBP1s mRNA was undetectable in adipose tissue and therefore we have no direct measurement of the activation of IRE1α in this tissue.

Independently of the UPR, ER stress induces an increase of lipid droplets [67]. The intracellular lipid accumulation is known to disrupt insulin pathway through the generation of lipid-derived toxic metabolites such ceramides and DAG [10], [11]. Intracellular lipids were not measured in this study. Consequently, we may not rule out an implication of intracellular lipids in the link between TLR4 dependent-ER stress and insulin resistance.

### Conclusion

TLR4 deficiency protects from obesity and glucose intolerance induced by a HFD as well as from ER stress in the main organs for glucose and lipid metabolism (skeletal muscle, liver and adipose tissue). Contrary to our hypothesis this phenomenon is not directly regulated by the NF-κB pathway but seems rather to be the indirect consequence of metabolic changes in TLR4^−/−^ mice which lead to less fat accumulation.
